# Supplementary material for: Unfair competition governs the interaction of pCPI-17 with myosin phosphatase (PP1-MYPT1)
Source: eLife. 2017 Apr 7;6:e24665. doi: 10.7554/eLife.24665 (PMC5441869; doi:10.7554/eLife.24665)
Supplement: Supplementary file 1. — DOI: http://dx.doi.org/10.7554/eLife.24665.020 [file elife-24665-supp1.pdf]

THEORETICAL METHODS

The analytic and computational methods are organized by figure number and grouped by the type of experiment being analyzed: using purified proteins, uterus extracts, or artery smooth muscle strips. All numerical calculations were performed using Mathematica (version 11). P, U, C, S, and I denote the unbound concentrations of MLCP phosphatase, PPU phosphatase, CPI-17, other substrates, and inhibitor, respectively. The prefix 'p' denotes the phosphorylated form. Total concentrations of bound plus unbound components are denoted by the subscript 'tot'. Parameters without superscripts refer to the pCPI-17/MLCP interaction; superscripts identify either other phosphatases or substrates, as implied by context. Unless otherwise indicated, experiments were conducted at (and parameters are for) 30°C.

Experiments with purified proteins

Figure 4: Measuring the pCPI-17/MLCP K_m by inhibitor competition

Because the pCPI-17/MLCP K_m is so small, it is difficult to measure accurately using standard velocity versus substrate concentration analysis. Instead, this value was determined with a modification of the method of Takai et al. (85) that uses competition with phosphatase inhibitors to determine ligand binding constants. This was a two-step process: First, we determined the okadaic acid (OA) and calyculin A K_is for MLCP using competition experiments with phosphosubstrates pS (either pMRLC, pMyBP, or pC-ERMAD) having large Michaelis constants, $K_m^{\mathrm{pS}} \gg pS_{tot}$, so that any uncertainties in the K_m^{pS}s were unimportant (see Figure 4 for concentrations and Table 2 for K_ms). Second, we computed the pCPI-17/MLCP K_m using these K_is to analyze measurements of the abilities of OA and calyculin A to inhibit dephosphorylation of pCPI-17 by MLCP. These experiments were performed at low MLCP concentration ($P_{tot} = 0.1$ nM) so as to sensitively report competition between the inhibitors and pCPI-17 for binding MLCP. In both cases we used the quasi-steady state equations

$$\begin{aligned} v &= k_{cat}^{\mathrm{pS}} \frac{pS_{tot}\ P}{K_m^{\mathrm{pS}}} \\ P &= P_{tot}/(1 + I/K_i + pS_{tot}/K_m^{\mathrm{pS}}) \\ I &= I_{tot}/(1 + P/K_i)\ , \end{aligned}$$

where v is the initial dephosphorylation rate, I is the inhibitor concentration, and we have approximated $pS \approx pS_{tot}$ because $P_{tot} = 0.1$ nM $\ll pS_{tot}$, so little phosphosubstrate was bound in all cases. These equations were analytically solved to determine v as a function of I_{tot}.

The best-fit value of the OA K_i was determined by nonlinear regression of $v(I)$ to the pMRLC and pMyBP dephosphorylation data. Analogous experiments to determine the calyculin A K_i^{cly}. The analyses gave $K_i^{\mathrm{OA}} = 20 \pm 2$ nM $(n = 7)$ and 0.13 nM $< K_i^{\mathrm{cly}} < 0.22$ nM $(n = 4)$. (K_i^{cly} could only be limited to a range because we only had a lower bound on $K_m^{\mathrm{pC\text{-}ERMAD}}$.)

The pCPI-17/MLCP K_m was then determined by nonlinear regression of $v(I)$ to the data for each inhibitor using the calculated K_i. Combining the OA ($K_m = 0.59 \pm 0.06; n = 8$) and calyculin A ($K_m \leq 0.42 \pm 0.04; n = 3$) estimates using Bayes' theorem gave $K_m = 0.48 \pm 0.03$ nM. This is an upper bound since the analysis assumes that both OA and calyculin A are purely competitive inhibitors. If inhibition is mixed, then the actual K_m is lower. The pCPI-17/MLCP k_{cat} for the MLCP batches used in these experiments was 0.044 sec^{-1} (OA experiments) and 0.053 sec^{-1} (calyculin A experiments). Averaging these with the values for the batches used in the experiments of Figure 2 – figure supplement 1 (0.08 sec^{-1}), Figure 3 (0.059 sec^{-1}), and Figure 5 (below; 0.062 sec^{-1}) gave the pCPI-17/MLCP $k_{cat} \sim 0.06 \pm 0.01$ sec^{-1} $(n = 4)$, which was used for subsequent analysis.

The values of the pC-ERMAD IC_{50}s shows that any contaminating phosphatases would have been inhibited at OA and

calyculin A concentrations much smaller than the pCPI-17 IC_{50}, and so would not have significantly affected the calculated K_m.

Figure 5: *In vitro* time-course of MLCP inhibition by pCPI-17

The analysis was complicated by two factors: (1) the small amount of contaminating phosphatase activity that remained even in the presence of a thio(p)CPI-17 concentration (50 nM) that completely inhibited MLCP, and (2) the slow degradation of MLCP that was noticeable as a slow decrease in the rate of pC-ERMAD dephosphorylation in the absence of pCPI-17. We accounted for these effects by including a background pC-ERMAD dephosphorylation rate, v^{bgd}, and an MLCP degradation rate, k^{deg} in the analysis. The time-dependent quasi-steady state equations in the presence of pCPI-17 are

$$d\,PO_4(t)/dt = \frac{k_{cat}^{\text{pS}}\,pS_{tot}}{K_m^{\text{pS}}}\,P + v^{bgd}$$
$$PO_4(t) = V\int_0^t d\,PO_4(t')/dt'\,dt'$$
$$P = P_{tot}\,e^{-k^{deg}t}/(1 + pC/K_m)$$
$$pC = pC_{tot}/(1 + pC/K_m)$$
$$\frac{d\,pC_{tot}}{dt} = -k_{cat}\,pC\,P/K_m\,,$$

where pS denotes pC-ERMAD, $V = 6\,\mu$M is the reaction volume, and $PO_4(t)$ is the amount of phosphate released by time t. The time dependence of v, P, pC_{tot}, and pC is implicit. Since $P_{tot} = 0.25$ nM $\ll 1.4\,\mu$M $= pS_{tot}$ and $pS_{tot} < 2.5\,\mu$M $< K_m^{\text{pS}}$, little pC-ERMAD was bound or dephosphorylated during the reaction. Therefore the equations incorporate the approximation $pS \approx pS_{tot} \approx$ constant. The pCPI-17/MLCP kinetic parameters were fixed at the values determined by previous experiments (Table 1). The other parameters were determined by (linear or nonlinear, as appropriate) regression from the relevant data. Solving the equations with these values gave the theoretical prediction for the time-course in the presence of pCPI-17 (red line, Figure 5).

Experiments with mouse uterus extracts

Figure 7 and Figure 7 – supplement 1: PPU has two components

These experiments were conducted with 890 nM ^{32}P-labeled pCPI-17 and extract total protein concentrations of $M = 0.093$ mg/ml (1:278 dilution) or $M = 0.047$ mg/ml (1:556 dilution). Because these concentrations were so low, the MLCP concentration was < 1 nM in both cases, so protection of pCPI-17 by MLCP was negligible and essentially all dephosphorylation was by other, more efficient, phosphatases (i.e., PPU). It is evident that most of the phosphatase activity was inhibited by OA with subnanomolar K_i, suggesting that this came from PP2A-, PP4-, and/or PP6-containing enzymes (84, 86). The inhibition curves were biphasic, thereby revealing the presence of a second component contributing a small amount of pCPI-17-dephosphorylating activity that was much less sensitive to OA inhibition. Therefore, the data were analyzed using a two-component model that included dephosphorylation by the PP2A-, PP4-, and PP6-like component, PPU^a, and an OA-resistant component, PPU^b

$$
\begin{aligned}
v &= (k_{cat}^{U^a}\, U^a / K_m^{U^a} + k_{cat}^{U^b}\, U^b / K_m^{U^b})\, pC \\
U^a &= U_{tot}^a / (1 + I / K_i^{U^a} + pC / K_m^{U^a}) \\
U^b &= U_{tot}^b / (1 + I / K_i^{U^b} + pC / K_m^{U^b}) \\
pC &= pC_{tot} / (1 + U^a / K_m^{U^a} + U^b / K_m^{U^b} \\
I &= I_{tot} / (1 + U^a / K_i^{U^a} + U^b / K_i^{U^b}) \,,
\end{aligned}
$$

where U^a and U^b denote the unbound PPU^a and PPU^b concentrations and v is the dephosphorylation rate. Nonlinear regression of three experiments at each dilution was used to determine $K_i^{U^b}$ and the total specific activities of PPU^a ($k_{cat}^{U^a}\, U_{tot}^a / M / K_m^{U^a}$) and PPU^b ($k_{cat}^{U^b}\, U_{tot}^b / M / K_m^{U^b}$) in the extract (Tables 1 and 2). This analysis showed that PPU^a and PPU^b contribute $\sim 85\%$ and $\sim 15\%$ of the total dephosphorylating activity, respectively. $K_i^{U^a}$ was too small to be determined from the data, but the PPU^a IC$_{50}$ provided the upper bound $K_i^{U^a} \leq$ IC$_{50} \sim 0.43$ nM.

The large value $K_i^{U^b} \sim 450$ nM precludes PPU^b being a PP2A-, PP4-, or PP6-type phosphatase. In addition, its activity is an order-of-magnitude greater than that predicted for MLCP. Therefore, we conclude that PPU^b is not MLCP. It might be a mixture of PP1 and other phosphatases even more resistant to OA leading to the large apparent inhibition constant.

Figure 7 – figure supplement 2: Determining K_m^U and $k_{cat}^{U^a}(30°\mathrm{C})/k_{cat}^{U^a}(0°\mathrm{C})$

Standard velocity versus substrate concentration experiments were performed with highly diluted uterus extracts so that the PPU kinetic constants could be determined without competition by other substrates or inhibitors present in the extracts (see discussion of Figure 8 – figure supplement 2, below). Since it was necessary to perform some of the uterus pCPI-17/extract dephosphorylation experiments at 0°C, we also determined kinetic constants at this temperature. Non-linear regression of the Michaelis-Menten formula gave the values reported in Table 2. While the experiments combined the effects of the two types of PPU, these values are close to those of PPU^a since PPU^b contributes only a small amount to the total PPU activity. The absence of biphasic structure in the curves indicates that $K_m^{U^b} \geq K_m^{U^a}$. Comparing the data at the two temperatures showed that $k_{cat}^U(30°\mathrm{C})/k_{cat}^U(0°\mathrm{C}) = 8.3 \pm 0.8$ ($n = 3$). This ratio and the approximate constancy of K_m^U with temperature is consistent with typical enzyme behavior (17).

Figure 8: Sequestration of pCPI-17 by MLCP in concentrated mouse uterus extracts and determination of [PPU^a]

To more closely mimic cellular protein concentrations, reactions were performed using highly concentrated uterus extract; the total protein concentration was $M = 17.5$ mg/ml. (While this was still $\sim 10\times$ lower than the estimated physiological concentration, it was adequate to test for sequestration of pCPI-17 by MLCP.) The pCPI-17 dephosphorylation reactions were performed at 0°C to limit adequately the fraction of substrate that was dephosphorylated. Based on the physiological MLCP concentration [~ 1 μM (3, 82)], the MLCP concentration in the reactions was estimated to be ~ 80 nM.

Determining [PPU$_{tot}^a$] from OA inhibition of unsequestered pCPI-17 dephosphorylation

The pCPI-17 concentration of 1.35 μM (black data, Figure 8) was in great excess over MLCP; so, in this case, only a negligible fraction was sequestered and almost all dephosphorylation was due to PPU. Since PPU^a accounts for most of the PPU activity and $K_i^{U^a} < 0.5$ nM (Figure 7), OA bound PPU^a almost stoichiometrically over the experimental range. Therefore, the IC$_{50}$ was close to half the total PPU^a concentration, U_{tot}^a. For best accuracy, U_{tot}^a was determined by nonlinear regression using the equations

$$\begin{aligned}
v &= (pC_{tot} - P_{tot})\,(k_{cat}^{\mathsf{U}^a}\,U^a/K_m^{\mathsf{U}^a} + k_{cat}^{\mathsf{U}^b}\,U^b/K_m^{\mathsf{U}^b}) \\
U^a &= U_{tot}^a/[1 + I/K_i^{\mathsf{U}^a} + (pC_{tot} - P_{tot})/K_m^{\mathsf{U}^a}] \\
U^b &= U_{tot}^b/[1 + I/K_i^{\mathsf{U}^b} + (pC_{tot} - P_{tot})/K_m^{\mathsf{U}^b}] \\
I &= I_{tot}/(1 + U^a/K_i^{\mathsf{U}^a})\ ,
\end{aligned}$$

where the kinetic parameters are for 0°C. Here we have approximated $pC \approx pC_{tot} - P_{tot}$ (because of the tight pCPI-17/MLCP binding and weak pCP-17/PPU binding), and the weak OA/PPU^b binding has been ignored. Nonlinear regression gave $U_{tot}^a/M = 8.9 \pm 0.9$ pmol/mg ($n = 3$). The total protein concentration in cells is typically $M = 135 - 200$ mg/ml (95), and we used the mean, $M_{phys} = 170$ mg/ml, with U_{tot}^a/M to estimate the physiological concentration to be [PPU$^a{}_{phys}$]$\sim 1.5\ \mu$M. Combining the value of U_{tot}^a/M with the results from Figures 7 and 7– figure supplement 1 gives $k_{cat}^{\mathsf{U}^a} = 41 \pm 6$ sec^{-1}. [Most of the uncertainty in this value comes from the determination of $K_m^{\mathsf{U}^a}$ (Figure 7 – figure supplement 1) and is irrelevant for the analysis of physiological results in Figure 9. That analysis is sensitive to the specificity constant $k_{cat}^{\mathsf{U}^a}/K_m^{\mathsf{U}^a}$, which is accurate within 5%.]

Dephosphorylation of pCPI-17 at low concentration is catalyzed by MLCP

When the pCPI-17 concentration was 10 nM (purple data, Figure 8), MLCP was in significant excess and the unfair competition model predicted that it would bind essentially all pCPI-17. Consequently, because the K_m (0.48 nM) is much smaller than the K_i (20 nM) for OA-inhibition, the model predicted that, as observed,an OA concentration much greater than the K_i would be required for inhibition. Moreover, the dephosphorylation rate in the absence of OA was ~ 0.008 nM/sec, in excellent agreement with the predicted MLCP dephosphorylation rate of $k_{cat}(0°\mathrm{C}) \times$ [pCPI-17]$_{tot} \sim 0.007$ nM/sec.

Figure 8 – figure supplement 1: Identifying the phosphatase activities against pC-ERMAD in uterus extracts

A priori, we allowed for the possibility that pC-ERMAD can be dephosphorylated by MLCP and other PP1-containing enzymes, PPU^a, and PPU^b by modeling the pC-ERMAD dephosphorylation rate as

$$v^{\mathsf{U}^b} = pS\,[k^{\mathsf{PP1}}/(1 + I/K_i^{\mathsf{PP1}}) + k^{\mathsf{U}^a}/(1 + I/K_i^{\mathsf{U}^a}) + k^{\mathsf{U}^b}/(1 + I/K_i^{\mathsf{U}^b})]\ ,$$

where pS denotes the unbound pC-ERMAD concentration, $k^{\mathsf{P}} \equiv k_{cat}^{\mathsf{P}}\,P/K_m^{\mathsf{P}}$ denotes the effective first-order activity of phosphatase 'P' against pC-ERMAD, and the K_is are given in Table 1. Because the extract was diluted $740\times$, the phosphatase concentrations were subnanomolar. Therefore, only small fractions of OA and pC-ERMAD were bound to enzyme, so $I \approx I_{tot}$ and $pS \approx pS_{tot}$. The PP1, PPU^a, and PPU^b activities were determined by nonlinear regression taking advantage of the fact that essentially all PPU^a was inhibited for [OA$_{tot}$]> 5 nM. This showed that MLCP and other PP1-containing enzymes contribute ~ 80% of the pC-ERMAD-directed phosphatase activity and that PPU^a and PPU^b each contribute ~ 10%.

Figure 8 – figure supplement 2: Competition from other substrates

Unidentified cellular substrates and inhibitors may bind MLCP and PPU, thereby sequestering these enzymes and reducing their dephosphorylation of pCPI-17 in cellular extracts and *in vivo*. This effect is insignificant at the dilute uterus extract concentrations used in the experiments of Figures 7 and 7 – supplement 1, but it can be important at physiological concentrations. In the presence of such competition, the dephosphorylation rate of substrate pS by phosphatase P (which here denotes either MLCP or PPU) is

$$v = k_{cat}^{\mathrm{pS}}\ pS\ P_{tot}\ /(1 + pS/K_m^{\mathrm{pS}} + \Sigma^{\mathrm{P}}) \tag{1a}$$

$$\Sigma^{\mathrm{P}} = \sum_i pS^{\mathrm{P},i}/K_m^{\mathrm{P},i}\ , \tag{1b}$$

where $pS^{\mathrm{P},i}$ and $K_m^{\mathrm{P},i}$ are the unbound concentration and Michaelis constant of substrate/inhibitor i of phosphatase P. Σ^{P}, the sum of all the terms arising from the binding of the phosphatase to the competing substrates and inhibitors, measures the strength of the competition.

We determined $\Sigma^{\mathrm{P}}_{phys}$, the competition parameter at physiological total protein concentration, by measuring the ability of uterus extracts that had been diluted to different total protein concentrations, M, to dephosphorylate a substrate that was known to be primarily dephosphorylated by phosphatase P. We modeled the variation of P_{tot} and Σ^{P} with M as

$$P_{tot}(M) = (M/M_{phys})\, P_{phys} \tag{2a}$$

$$\Sigma^{\mathrm{P}}(M) \approx (M/M_{phys})\, \Sigma^{\mathrm{P}}_{phys}\ . \tag{2b}$$

The first equation is precise, since total concentrations vary linearly with M. While the second equation is an approximation that ignores concentration-dependent changes in the ratio between the unbound and total $\mathrm{S}^{\mathrm{P},i}$ concentrations, we found that it well-fit either the entire data range (panel A) or the high-concentration region (i.e., $M \geq 0.01\ M_{phys}$; panel B), which was adequate for determining $\Sigma^{\mathrm{P}}_{phys}$.

Since pCPI-17 at high concentrations is dephosphorylated almost exclusively by PPU (see Figure 8), it provided a good substrate for determining $\Sigma^{\mathrm{U}}_{phys}$. Experiments were performed with ^{32}P-labeled pCPI-17 at 1.25 μM and varying uterus extract concentrations with $M \leq 45$ mg/ml, the highest concentration that was technically feasible. Since $K_m \ll$[pCPI-17$_{tot}$]$\ll K_m^{\mathrm{U}}$, MLCP, but not PPU, sequestered pCPI-17; therefore, we approximated $pS \approx pS_{tot} - (M/M_{phys})$ [MLCP$_{phys}$]. We then used nonlinear regression of Eqs. (1) and (2) to the data and determined $\Sigma^{\mathrm{U}}_{phys} = 24 \pm 6$ (panel A).

Since pC-ERMAD is primarily dephosphorylated by PP1-type enzymes (see Figure 8 – supplement figure 1), it was a good substrate for determining $\Sigma^{\mathrm{PP1}}_{phys}$. Since pS/K_m^{pS} was small, it was ignored in Eq. (1a). Nonlinear regression using Eqs. (1) and (2) with the data with $M \geq 0.01\ M_{phys}$ with an adjustment for the additional change in phosphatase activity as $M \to 0$ gave a best-fit of $\Sigma^{\mathrm{PP1}} = 15 \pm 2$ (panel B). We assumed that this was representative of competition for MLCP and used this value as $\Sigma^{\mathrm{MLCP}}_{phys}$.

$\Sigma^{\mathrm{U}}(M)$ and $\Sigma^{\mathrm{MLCP}}(M)$ were ~ 2 at the extract concentrations used in the experiments of Figure 8, but this did not affect the the computation of [PPU^{a}], or the predicted dephosphorylation rate at low pCPI-17 concentration. In contrast, these estimates do play a significant role in calculating the time-courses in Figure 9, as described in the next section.

Experiments with rabbit artery smooth muscle strips

Figure 9: Physiological activation of MLCP and dephosphorylation of pCPI-17 upon vasodilation

Kitazawa et al. (53) determined that the phosphorylation of CPI-17 decreased by $46 \pm 8\%$ with a half-life of about 10 sec following addition of SNP, which initiated the transition from vasocontraction (vc) to vasodilation (vd). Cotemporally, MRLC phosphorylation decreased from 0.47 ± 0.02 to 0.27 ± 0.06 (their Figure 4).

The change in MLCP activity was not measured, but we inferred this from the experimental change in the phosphorylation level of MRLC. In principle, the MRLC phosphorylation level depends on both the MLCK and MLCP activities as determined by the equilibrium relationship

$$k_{cat}^{\mathrm{MLCK}} \frac{[\mathrm{MLCK}]\,[\mathrm{MRLC}]}{K_m^{\mathrm{MLCK}}} = k_{cat}^{\mathrm{MLCP}} \frac{[\mathrm{MLCP}]\,[\mathrm{pMRLC}]}{K_m^{\mathrm{MLCP}}}\ , \tag{3}$$

where the kinetic constants are for the enzyme identified by superscript and the MRLC or pMRLC substrate as appropriate. However, Woodsome et al. (96) found that (1) the sensitivity of MRLC phosphorylation to increased Ca^{2+} concentration in a variety of smooth muscle tissues correlated with the expression ratio of CPI-17 to MLCP concentrations, not with MLCK concentration, and (2) activation of PKC (and the resultant increase in CPI-17 phosphorylation) was sufficient to induce both MRLC phosphorylation and vasocontraction. These facts motivated the working hypothesis that the observed decrease in MRLC phosphorylation was due primarily to increased MLCP activity. In this case, Eq. (3) implies that $P(\text{vd})/P(\text{vc}) = 2.4$, and we used this value in the simulations described below. We also performed simulations that either used the previous experimental result $P(\text{vd})/P(\text{vc}) = 3$ (30) or that ascribed some of the reduction in MRLC phosphorylation during vasodilation to decreased MLCK activity (thereby implying a smaller MLCP activation). These controls showed that the computed half-lives for pCPI-17 dephosphorylation and MLCP activation were not highly sensitive to the value used for $P(\text{vd})/P(\text{vc})$.

The simulations of the time-dependent changes in CPI-17 phosphorylation, MRLC phosphorylation, and MLCP activity following the addition of SNP were performed using the experimental values from Ref. (53), prior estimates of the total MLCP concentration [$\sim 1\ \mu$M (3; 82)] and the total concentration of CPI-17 plus pCPI-17 [7 μM (96, 52)], and the parameters measured in this study (Tables 1 and 2). These simulations used the time-dependent quasi-steady state equations

$$pC = pC_{tot}/(1 + P/K_m + U/K_m^{\mathrm{U}})$$
$$P = P_{tot}/(1 + pC/K_m + \Sigma^{\mathrm{P}})$$
$$U = U_{tot}(1 + pC/K_m^{\mathrm{U}} + \Sigma^{\mathrm{U}})$$
$$d\,pC_{tot}/dt = -k_{cat}\, pC\, P/K_m - k_{cat}^{\mathrm{U}}\, pC\, U/K_m^{\mathrm{U}} + k_+\, C_{tot}\ ,$$

where time-dependence is left implicit and $pC_{tot} + C_{tot} = 7\ \mu$M is constant. k_+ denotes the first-order CPI-17-phosphorylating activity and is assumed to change rapidly from $k_+(\text{vc})$ to $k_+(\text{vd})$ upon addition of SNP at $t = 0$. Along with the experimental $pC_{tot}(\text{vc})/pC_{tot}(\text{vd}) = 1.85$ and computed $P(\text{vd})/P(\text{vc}) = 2.4$, the first three equations applied both in vc and vd provide eight equations for the eight unknowns (pC, pC_{tot}, P, and U in vc and vd). The solution is $P(\text{vc}) = 15$ nM, $P(\text{vd}) = 37$ nM, $pC(\text{vc}) = 24$ nM, $pC(\text{vd}) = 5.5$ nM, $pC_{tot}(\text{vc}) = 0.78\ \mu$M, and $pC_{tot}(\text{vd}) = 0.42\ \mu$M, $U(\text{vc}) = 65$ nM, and $U(\text{vd}) = 65$ nM. Using the stationary conditions $d\,pC_{tot}/dt = 0$ (i.e., corresponding to the vc and vd equilibrium states), we calculated $k_+(\text{vc}) = 0.0073\ \text{sec}^{-1}$ and $k_+(\text{vd}) = 0.0038\ \text{sec}^{-1}$; i.e., the CPI-17-directed kinase activity decreased about two-fold from vc to vd. We then numerically integrated the equations starting from $t = 0$ with $k_+ = k_+(\text{vd})$ and using $P(\text{vc})$, $pC(\text{vc})$, $pC_{tot}(\text{vc})$, and $U(\text{vc})$ as $t = 0$ boundary conditions. This computation gave $pC(t)$ and $P(t)$ over the experimental time interval. The time-dependent level of MRLC phosphorylation was then computed using Eq. (3).

These functions are plotted in Figure 9. The plots for the hypothetical cases in which only MLCP or only PPU acts on pCPI-17 were computed in the same manner but with either k_{cat}^{U} or k_{cat} set to zero, respectively. The half-lives for activation of MLCP were 10.4 sec (MLCP plus PPU), 11.6 sec (MLCP only), and 98 sec (PPU only). To test the sensitivity of the results to the estimated MLCP concentration, the calculations were repeated with its lower (0.7 μM) and upper (2 μM) experimental bounds (3, 82).

The control simulation using the prior, independent, experimental measurement of MLCP activation, $P(\text{vd})/P(\text{vc}) = 3$ (30), confirmed that the experimental data could only be explained by the MLCP + PPU and MLCP-only models, not by the PPU-only model. (In this latter case, the half-life for pCPI-17 dephosphorylation in the PPU-only model was ~ 80 sec.) And simulations that included a decrease in MLCK activity during the vc→vd transition actually resulted in an increase in the computed half-life of MLCP activation when only PPU was assumed to dephosphorylate pCPI-17. We performed additional

control computations to test the effect of two-fold variations of other parameters (i.e., Σ^{MLCP}, Σ^{U}, and K_m^{U}). The simulation and controls confirmed that dephosphorylation by PPU alone could not explain the experimental data, supporting the hypothesis that dephosphorylation of pCPI-17 by MLCP is needed to explain the experimental measurements of Ref. (53).
